# Morphological and Molecular Characterization of *Lasiodiplodia theobromae* Causing Stem Gummosis Disease in Rubber Trees and Its Chemical Control Strategies

**DOI:** 10.3390/microorganisms13071586

**Published:** 2025-07-05

**Authors:** Chunping He, Jinjing Lin, He Wu, Jinlong Zheng, Yong Zhang, Yu Zhang, Zengping Li, Yanqiong Liang, Ying Lu, Kexian Yi, Weihuai Wu

**Affiliations:** 1 Key Laboratory of Integrated Pest Management on Tropical Crops, Environment and Plant Protection Institute, Ministry of Agriculture and Rural Affairs, Chinese Academy of Tropical Agricultural Science, Haikou 571101, China; hechunppp@163.com (C.H.); zhengjinlong_36@163.com (J.Z.); yanqiongliang@126.com (Y.L.); ytluy2010@163.com (Y.L.); 2 College of Plant Science and Technology, Huazhong Agricultural University, Wuhan 430070, China; 18870357309@163.com; 3 Sanya Institute of Breeding and Multiplication, School of Tropical Agriculture and Forestry, Hainan University, Haikou 570228, China; wh19990709@163.com (H.W.); yuzhang_rain@163.com (Y.Z.); lzping301155@126.com (Z.L.); 4 Honghe Tropical Agiculture Science Institute of Yunnan, Hekou 661300, China; zy170315@126.com; 5 Sanya Research Institute of Chinese Academy of Tropical Agricultural Sciences, Sanya 572025, China

**Keywords:** *Lasiodiplodia theobromae*, stem gummosis disease, rubber tree, pathogenicity, morphology, phylogenetic analysis, fungicides

## Abstract

Rubber tree (*Hevea brasiliensis* Muell. Arg.) is a major tropical cash crop in southern China, with Hainan and Yunnan provinces being the main planting areas. In July 2023, bark cracking and gumming were observed on the trunks of mature rubber trees in Haikou City, Hainan Province, leading to xylem rot, which severely impacted the healthy growth of the rubber trees. The present study was conducted to confirm the pathogenicity of the patho-gen associated with stem gummosis disease, characterize it using morphological and mo-lecular tools, and devise field management strategies. Pathogenicity testing showed that this strain induced symptoms similar to those of natural outdoor infestation. Based on morphological study and molecular analyses of internal transcribed spacer (*ITS*), transla-tion elongation factor 1 alpha (*TEF1-α*), and β-tubulin 2 (*TUB2*) sequences, the causal agent was identified as *Lasiodiplodia theobromae*. Field trials demonstrated that an inte-grated fungicide approach—combining trunk application of Bordeaux mixture with root irrigation using citric acid–copper 6.4% + chelated copper-ammonium 15% at both 0.1% and 0.2% concentration—effectively suppressed stem gummosis disease incidence in rub-ber trees. To the best of our knowledge, this is the first report of *L. theobromae* causing stem gummosis on rubber tree in China. The findings of this study can provide valuable infor-mation for the management strategies and understanding of this disease.

## 1. Introduction

*Hevea brasiliensis* (Willd. ex A.Juss.) Muell. Arg. is an angiosperm plant belonging to the genus *Hevea* in the Euphorbiaceae family, which is a perennial tropical deciduous large tree; it has the widest distribution, is native to the Amazon Basin of Brazil in South America, and is currently cultivated on approximately 13 million hectares of land worldwide [1,2]. This variety of plant was introduced to Asia at the end of the 19th century [3]. It was introduced to China from Singapore in 1904 and has a history of one hundred years, and is mainly distributed in tropical and subtropical regions such as Hainan, Yunnan, Guangxi, Guangdong, and Taiwan [4]. Among the various plants, *Hevea brasiliensis* is considered the most economically valuable because it is the plant with the highest latex content, and its extracted latex is the main source of natural rubber worldwide [5]. Natural rubber exhibits excellent properties such as high elasticity, high strength, insulation, wear resistance, medium resistance, corrosion resistance, good sealing performance, and weather resistance. After more than a century of development, there are now over 7,000 types of rubber products, which are widely used in daily life, industrial and agricultural production, transportation, electronics and telecommunications, aerospace, and military applications. Due to its irreplaceable role, natural rubber is a critical industrial raw material and strategic resource [6,7,8].

Rubber trees are susceptible to various phytopathogenic fungi during planting, causing numerous diseases at different stages of growth. Common diseases of rubber trees are powdery mildew, anthracnose, root rot, stem canker, and brown bark. For many years, rubber trees have been affected by root diseases caused by *Phellinus noxius*, *Rigidoporus microporus*, and *Ganoderma philippii* [9]. In China, *Colletotrichum siamense* and *C. australiensis* are the main pathogens causing anthracnose disease in rubber trees [10]. *Fusarium solani* has been identified as the causative pathogen of trunk and branch gummosis disease, affecting rubber trees in Baisha City, Hainan Province [11]. These diseases have caused significant economic losses to the natural rubber industry.

Among many fungal genera that are harmful to plants, *Lasiodiplodia* is one of the most well-known. *Lasiodiplodia* species are members of the *Botryosphaeriaceae*, which is widely distributed in tropical and subtropical regions, and has been associated with appromimately 500 hosts, which has seriously affected the growth of agricultural and forestry plants [12], which are known for their ability to cause devastating diseases such as leaf blight [13], trunk gummosis [14,15], dieback [16,17,18], stem canker [19], and stem rot [20]. Diseases in rubber trees caused by *L. theobromae* have been reported in countries such as Brazil, India, Malaysia, Myanmar, Nigeria, Papua New Guinea, the Philippines, and Thailand [21]. In China, there have been reports that *L. theobromae* can cause stem blue discoloration [22], leaf spots [21], root necrosis [23], and diebark [24] in rubber trees. Zhao et al. discovered that *L. theobromae* could cause severe mold and stain of newly felled rubber wood within just 2–3 days in two southern Chinese provinces (Yunnan and Hainan), significantly reducing its processability and utilization value, leading to substantial economic losses [22]. During 2015–2016, Cai et al. reported the first occurrence of *L. theobromae*-induced leaf spot disease in eight rubber tree nurseries across Guangdong, Hainan, and Yunnan, though infections were sporadic at the time [21]. In 2017, Hu et al. identified *L. theobromae* as the causal agent of rubber tree dieback in Hainan, affecting approximately 266.6 hectares of plantations. The disease incidence ranged from 1.9% to 50%, with plant mortality rates between 0.4% and 40% [24]. From 2016 to 2017, Jiang et al. documented *L. theobromae*-associated rapid decline disease in eight major rubber plantations in Yunnan, characterized by sudden and widespread tree death. This disease poses an extremely serious threat to rubber production due to its aggressive nature [23]. In addition, there have been no research reports on the damage caused by this pathogen to live rubber trees in China.

In July 2023, typical symptoms of gummosis were observed on 7-to-8-year-old *Hevea brasiliensis* plants in the Natural Rubber Science Museum of the Chinese Academy of Tropical Agricultural Sciences (CATAS), Haikou, China (19°59′1″ N, 110°19′26″ E). The condition had an incidence rate of 18%, and as the disease developed, continuous white latex exudation from the tree stems was observed, accompanied by cracking, ulceration, and xylem necrosis, severely affecting both healthy growth and the ornamental value of the rubber trees. The main objective of this study was to identify the pathogenic species responsible for rubber tree gummosis through pathogenicity tests on representative isolates, and morphological characterization combined with multi-locus phylogenetic analysis. The efficacy of fungicides in managing stem gummosis disease in rubber trees was evaluated.

## 2. Materials and Methods

### 2.1. Sample Collection and Pathogen Isolation

In July 2023, samples ranging from 10 cm to 15 cm in length were collected from the diseased stems of rubber tree plants exhibiting the characteristic signs of gummosis from surrounding the Natural Rubber Science Museum of the Chinese Academy of Tropical Agricultural Sciences in Haikou, China (19.9836° N, 110.3239° E). They were packed in a sterilized zipper polybag and brought to the laboratory of CATAS Institute of Environment and Plant Protection; then, the diseased samples were rinsed with water and cut into several small pieces 3 mm × 3 mm in size; the specimen was treated via immersion in 75% ethanol for 20 s and in 2% sodium hypochlorite for 10 s, rinsing three times with sterile distilled water, and then excess water was removed with sterile filter paper and placed on potato dextrose agar (PDA) medium amended with tetracycline (10 mg mL^−1^). These inoculated plates were kept at 28 °C in darkness for 2 days. The mycelia grown in the samples were transferred to fresh PDA and pure cultures were obtained by single-spore isolation.

### 2.2. Koch’s Postulate Test

Pathogenicity tests were conducted on 3-year-old healthy potted seedlings of *Hevea brasiliensis* (cultivar Reyan 73397), obtained from the Rubber Research Institute of the Chinese Academy of Tropical Agricultural Sciences in Haikou, China. The stems were surface-disinfected with 75% ethanol, then punctured with a sterilized needle and inoculated with two mycelial disks (5 mm in diameter) taken from the edge of 6-day-old fungal colonies cultured at 28 °C. The disks were placed mycelium-side down onto each wound. Sterilized PDA blocks were used as the control, with three replicates per treatment. The inoculated seedlings were kept in a natural environment, with moisture preserved using wet cotton and sealed with plastic film. Disease symptoms development at the inoculation sites were recorded daily. Fungi were re-isolated from stems showing lesions and their morphological characterizations were compared with pathogens which were previously isolated from diseased *H. brasiliensis* stems.

### 2.3. Morphological Identification

The fungal pathogen was picked up with a needle and placed on a clean microscope slide; water was dropped onto it and the slide was re-covered, and we observed the fungal morphology under a Ni-E Biomicroscope (Nikon, Ōtawara, Japan). The size and shape of colonies, conidiophores, and conidia were analyzed. The size of the spores of the fungal isolates was measured (*n* = 60).

### 2.4. DNA Extraction, PCR Amplification, and Sequencing

Fungal isolates were cultured on PDA at 28 °C for 10 days. The mycelium was harvested and finely ground into powder in liquid nitrogen. The fungal genomic DNA was extracted using a fungal DNA extraction kit (Omega Bio-tek, Inc., Beijing, China), and the presence of total DNA was detected by 1% (*w*/*v*) agarose gel electrophoresis stained with GoldViewII (Beijing Solarbio Science & Technology, Beijing, China) and viewed under transmitted ultraviolet light using Alliance 6.7 (UVItec Ltd., Cambridge, UK).

Internal transcribed spacer (ITS), translation elongation factor (*TEF1-α*), and beta-tubulin (*TUB2*) were amplified with ITS1/ITS4 [25], EF1- 688F/EF1- 1251R [26], and Bt2a/Bt2b [27] primers, respectively. The PCR reaction systems were all 25 μL, containing 2 × Taq PCR Master Mix (12.5 μL), DNA template (1 μL), each primer (1 μL), and ddH_2_O (9.5 μL). PCR was performed in a Mastercycler (Eppendorf, Germany). The PCR amplification program was as follows: initial denaturation at 95 °C for 5 min, denaturation at 95 °C for 30 s, annealing for 45 s (ITS: 55 °C, *TEF1-α*: 59 °C, *TUB2*: 62 °C), extending at 72 °C for 33 cycles, and extending at 72 °C for 10 min. PCR amplification products were detected by 1.0% agarose gel electrophoresis and sequenced by Liuhe Huada Technology Co., Ltd. (Beijing, China).

### 2.5. Sequence Alignment and Phylogenetic Analysis

The sequencing data were submitted to the NCBI database, and an accession number was assigned to the work. Sequence homology for the ITS, *TEF-1α*, and *TUB2* regions were analyzed using BLAST 2.14.0 in the NCB database (https://blast.ncbi.nlm.nih.gov/Blast.cgi accessed on 25 November 2023). The ITS, *TEF-1α*, and *TUB2* sequences used for phylogenetic analysis are listed in Table S1. Multiple sequence alignment was performed using the Clustal W method in MEGA 5.0, and phylogenetic trees were constructed using the Maximum Likelihood (ML) method with 1000 bootstrap replicates. The phylogenetic tree was generated based on the concatenated sequences of ITS, *TEF-1α*, and *TUB2*.

### 2.6. Field Experiment

From August to September 2023, an outdoor field experiment was conducted to control gummosis in rubber trees. The treatment protocol consisted of two integrated measures: (1) Scraping off coarse bark to expose infected tissues, followed by the topical application of inorganic copper-based fungicide to trunk lesions. Freshly prepared Bordeaux mixture (1:1:10, copper sulfate:lime:water) was applied to trunk surfaces up to 4 m in height. (2) Concurrent root irrigation with fungicidal solution. Citric acid–copper 6.4% + chelated copper-ammonium 15% at both 0.1% and 0.2% concentration was applied as soil drench around the base of affected trees. Treatments were administered at 7-day intervals for three consecutive applications, and the growth conditions of diseased plants were observed after fungicide application.

## 3. Results

### 3.1. Disease Symptoms and Pathogen Morphological Identification

**Figure 1:**
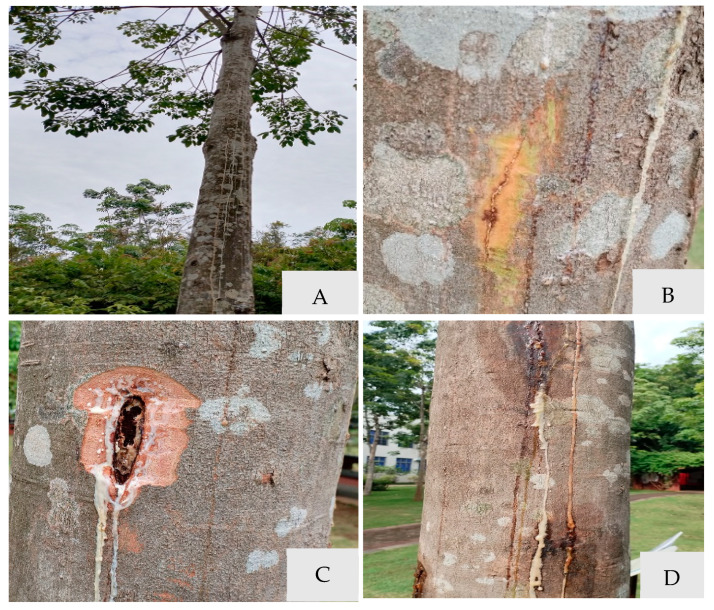
Symptoms of stem gummosis of *Hevea brasiliensis* in the field. (**A**) Evident stem blotch and cankers on the stem. (**B**) Bursting bark and flow white latex on the stem in the initial stage. (**C**) Xylem of the flowing gum extended longitudinally in the later stages. (**D**) White and brown glue line in the outer stem.

In the Natural Rubber Science Museum of the Chinese Academy of Tropical Agricultural Sciences (CATAS), 18% (5/27) of rubber trees plants showed stem gummosis disease from July to October 2023 in Haikou, China. Among the six rubber tree varieties cultivated (Haiken 1, Reken 628, Reyan 8-79, Reyan 73397, PR107 and RRIM600) for display in the science museum, only Reken 628 and Reyan 73397 were severely affected, with incidence rates of 100% (2/2) and 75% (3/4), respectively. The symptoms of disease on the stems initially were observed bursting of the bark and flow of the white latex (Figure 1A,B). As the disease progressed, xylem necrosis and solidification of the internal latex blocked the bast (Figure 1C). Subsequently, the latex gradually solidified, forming a brown glue line (Figure 1D).

**Figure 2:**
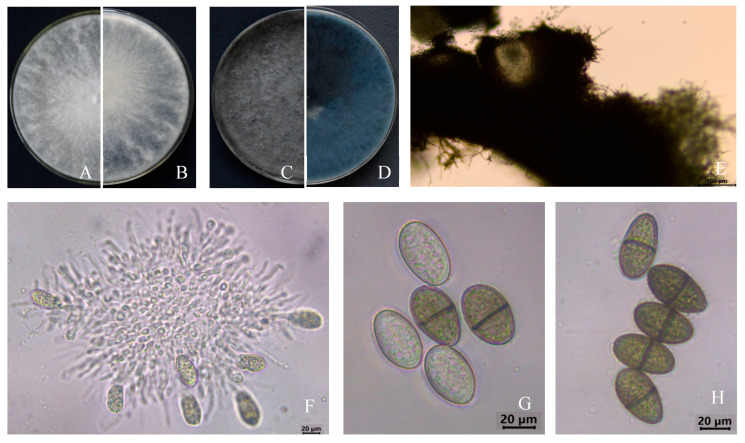
Morphological characteristics of fungi isolated (J2) from *Hevea brasiliensis*. (**A**,**B**) Colony morphology on PDA from above (left) and reverse (right) after 3 d. (**C**,**D**) Colony morphology on PDA from above (left) and reverse (right) after 10 d. (**E**) Pycnidium on PDA. (**F**) Young conidia. (**G**) Immature conidia. (**H**) Mature conidia. Scale bars: (**E**) = 100 µm; (**F**–**H**) = 20 µm.

Eleven fungal isolates (named J1–J11) with identical colony morphology, conidia morphology, and size were obtained from the infected stems tissues by the tissue separation method. The representative strain J2 with good growth was employed for morphology study. The pure culture of colonies grew rapidly on the PDA medium, and after 3 d of incubation at 28 °C, were round, with a neat edge and radial growth, white at the beginning of growth (Figure 2A,B), later aging to blue-black and to black (Figure 2C,D). The pycnidium was compressed, flat-globose, and black (Figure 2E). The paraphyses were hyaline, upward, and cylindrical, and the conidia cells were round, colorless, and hyaline (Figure 2F). There were two forms of conidia: Immature conidia were ovoidal to ellipsoidal, single-celled, colorless, and transparent (Figure 2G). Mature conidia were dark brown, double-celled with a septum in the center, and measured (18.60~25.07) μm × (10.57~14.44) μm in size (*n* = 60) (Figure 2H). The morphological characteristics of the isolates matched those of *L. theobromae* [26].

### 3.2. Pathogenicity Test

**Figure 3:**
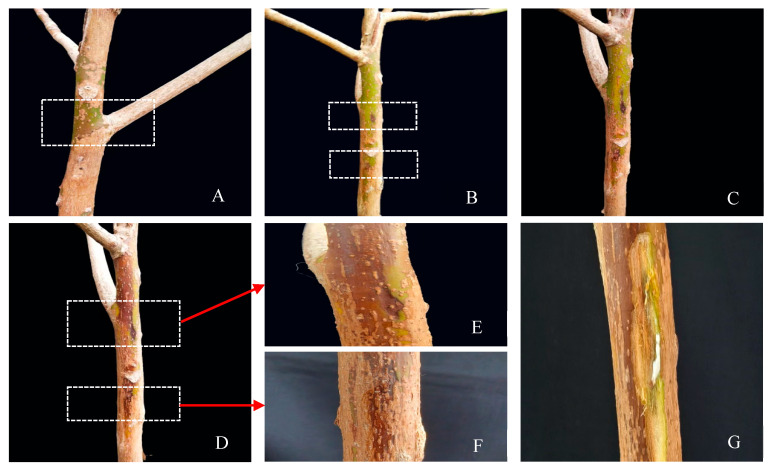
The symptoms of isolate J2 artificially inoculated on live rubber tree seedlings. (**A**) Control stem (inoculated with pure PDA plugs). (**B**) Inoculated with J2 isolate mycelial plugs for 2 d. (**C**) Inoculated with J2 isolate mycelial plugs for 5 d. (**D**) Inoculated with J2 isolate mycelial plugs for 8 d. (**E**,**F**) A close-up of stem lesions with discoloration and brown spots is indicated with a white box. (**G**) Bark removal of discoloration areas in the same seedings reveals brown necrotic in the xylem of the stem.

In late July 2023, a representative fungal strain J2 was selected for pathogenicity determination. The fungus was inoculated onto 3-years-young rubber tree seedlings with three replicates per treatment. Two days post-inoculation for the J2 isolate, brown lesions emerged at the puncture sites on the stems of the seedlings (Figure 3B). By the fifth day, the lesions had expanded further (Figure 3C), and by the eighth day, they had coalesced, forming extensive necrotic areas that spread upwards along the stems (Figure 3D–F). Upon dissection, the underlying xylem exhibited pronounced browning and necrotic symptoms (Figure 3G). Notably, all inoculated seedlings displayed symptoms consistent with field-infected plants, whereas control seedlings remained asymptomatic throughout the observation period (Figure 3A). To confirm Koch’s postulates, the re-isolated fungus from the diseased stems was consistent with the inoculated isolate J2 and shared the same morphological characteristics. Therefore, the J2 isolate was determined to be the pathogenic fungus responsible for rubber tree stem gummosis disease.

### 3.3. Sequence, Identification of Pathogen Species, and Phylogenetic Analysis

**Figure 4:**
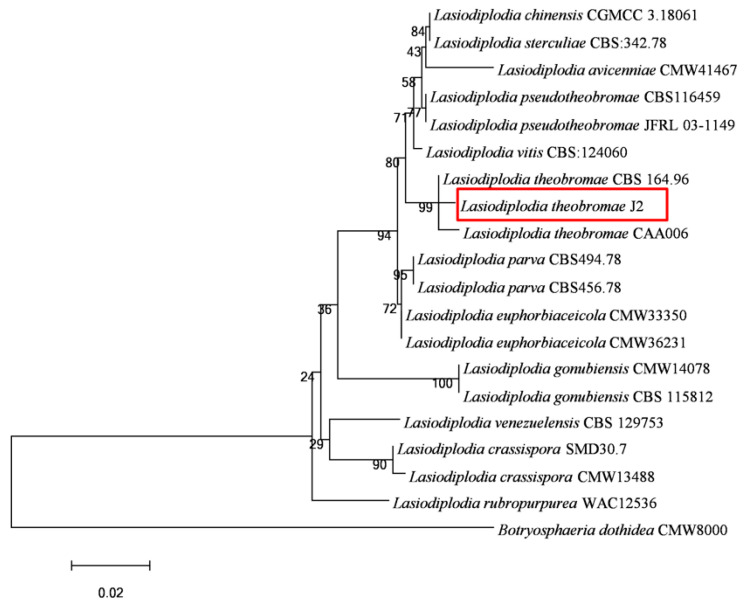
The maximum likelihood (ML) tree generated from the combined sequences of ITS, *TEF1-α*, and *TUB2* genes, with *Botryosphaeria dothidea* CMW8000 used as an out-group. Bootstrap support values with 1000 replications are shown at the nodes of the branches.

Amplicons of 519 bp, 520 bp, and 407 bp were obtained by amplifying genomic DNA from J2 isolates using ITS, *TEF-1α*, and *TUB2* primers, respectively. The assembled sequences were submitted to NCBI GenBank with accession numbers OR733551 (*ITS*), OR754359 (*TEF1-α*), and OR754360 (*TUB2*), respectively. The ITS sequence of the fungal isolate J2 revealed more than 99% homology with *L. theobromae* in the GenBank of BLAST search. Sequences downloaded from NCBI were then used to carry out cluster analysis. The *Botryosphaeria dothidea* isolate CMW8000 was selected as an out-group (Table 1). Multilocus phylogenetic analysis further showed that the fungal isolate J2 was clustered in the same branch with the reference isolates of *L. theobromae*, supported by a 99% bootstrap value (Figure 4). Therefore, the isolate was confirmed to be *L. theobromae* on the grounds of molecular identification.

### 3.4. Preliminary Fungicide Observations

**Figure 5:**
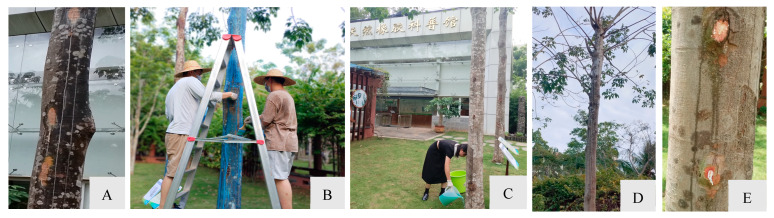
Integrated control measure. (**A**) Scraped infected bark. (**B**) Trunk coating of Bordeaux mixture. (**C**) Root irrigation using fungicide. (**D**) Recovery of treated plants (18 months). (**E**) Stem regeneration profile (Scrape off coarse bark,18 months).

Upon detecting stem gummosis in rubber trees, an integrated control measure was implemented (Figure 5). This involved topical application of an inorganic copper-based fungicide (Bordeaux mixture) to the affected trunk areas (Figure 5A,B), combined with root irrigation using citric acid–copper 6.4% + chelated copper-ammonium 15% at both 0.1% and 0.2% concentration (Figure 5C). After 18 months of continuous monitoring, the initial gummosis symptoms in infected trees gradually disappeared and the treated plants grew well (Figure 5D,E). The treatment effectively suppressed symptom progression and demonstrated strong disease control, with no new disease incidence observed.

## 4. Discussion

The accurate identification of the pathogen is crucial for the effective management and control of the disease. In the genus *Lasiodiplodia*, *L. theobromae* is taxonomically similar to *L. pseudotheobromae*, but they differ subtly in their conidia, with *L. theobromae* having relatively larger, rounded-end conidia. Additionally, they also clustered in different branches in the evolutionary tree [21]. The isolate in this study was morphologically similar to *L. theobromae* and clustered in a branch with it. Based on morphological characteristics, multi-gene phylogenetic analyses, and pathogenicity assessments, the isolates obtained from *H. brasiliensis* stems exhibiting gummosis were identified as *L. theobromae*. To our knowledge, this is also the first report of stem gummosis on *H. brasiliensis* associated with *L. theobromae* in China.

*L. theobromae* is a plant pathogen with a broad host range, primarily infecting woody plants. It affects various tissues including buds, young shoots, and trunks of numerous tree species such as *Albizia*, pear, apple, peach, mango, cashew, avocado, citrus, cocoa, eucalyptus, rose, nectarine, pine, neem, and other species. The infection can lead to shoot blight, branch dieback, gummosis, root rot, and stem canker [15,16,19,23,28,29,30,31,32,33,34,35,36,37]. According to domestic and international literature, *L. theobromae* can cause branch blight in *Aquilaria sinensis* [38], branch blight in *Cinnamomum cassia* [39], leaf blight in *Aloe vera* [40], gummosis in *Mangifera indica* [29], grapevine trunk ulcer [41,42,43], root rot in *Morus* spp. [44], fruit rot in *Artocarpus heterophyllus* [45], leaf blight is [46], stem canker in *H. brasiliens* [47], and rubberwood blue stain [48]. Moreover, in China, leaf spot [21], dieback [24], root rot [23], and rubberwood blue stain [22], respectively, were reported in *H. brasiliensis* caused by *L. theobromae*, but no cases of stem gummosis in *H. brasiliensis* were caused by this pathogen.

The management of diseases caused by *Lasiodiplodia theobromae* requires a multifaceted approach, as currently, very few fungicides are advised for management of diseases. For instance, mancozeb has demonstrated significant efficacy against *L. theobromae*, the causative agent of sweet-potato root rot [49]. Similarly, carbendazim, fludioxonil, and difenoconazole exhibit inhibitory effects on *L. Theobromae* associated with jackfruit fruit rot [50]. Other fungicides, including captan, tebuconazole and propiconazole, and carbendazim, show strong antifungal activity, with mycelial inhibition rates exceeding 98% against *L. Theobromae* in mulberry root rot [51]. However, Bhadra et al. [52] reported that Bavistin (carbendazim) and Dithane M-45 (mancozeb) had almost no inhibitory effect on *L. theobromae*, highlighting inconsistencies in fungicide performance. These studies indicate that strains from different sources also have significant differences in sensitivity to the same fungicide. To develop management strategies for stem gummosis disease in rubber trees, the efficacy of fungicides in controlling this disease was evaluated. This study found that trunk application of Bordeaux mixture combined with root irrigation using citric acid–copper 6.4% + chelated copper-ammonium 15% at both 0.1% and 0.2% concentration effectively controlled stem gummosis disease in rubber trees. Currently, the early application of chemical fungicides remains the primary approach for controlling crop diseases [53]. However, excessive use of fungicides not only causes environmental pollution and threatens consumer health but may also lead to the development of pathogen resistance. Therefore, the development of alternative and sustainable control strategies is urgently needed [54]. In the future, an integrated disease management system should be established, incorporating ecological cultivation techniques, development of biocontrol agents, breeding of disease-resistant varieties, screening of botanical pesticides, and application of highly effective and low-toxicity fungicides, to achieve coordinated control through multiple approaches and minimize reliance on chemical pesticides.

Infection by *L. theobromae* in rubber trees manifests as bark swelling and fissuring with white latex exudation. As the disease progresses, the exudate forms brown gum streaks, accompanied by stem cracking, canker formation, and xylem necrosis, ultimately leading to growth retardation and substantial latex yield losses. During the investigation of rubber trees cultivated at the Natural Rubber Science Museum (CATAS, Haikou), it was found that rubber tree gummosis mainly occurs during the high temperature and typhoon season from July to October. With prolonged exposure to sunlight and mechanical damage caused by typhoons, it is easy to create conditions for the invasion and spread of pathogens. This study focused on a newly emerging disease, rubber tree stem gummosis disease in Hainan. Through morphological characterization and molecular identification using three sets of specific primers, the pathogen was identified for the first time as *Lasiodiplodia theobromae*. This diagnostic system significantly improves the accuracy of pathogen detection, providing critical technical support for early disease diagnosis and scientific quarantine measures. Given the significant harm of this disease to the natural rubber industry, growers should attach great importance to it in production and further strengthen the monitoring and prevention of this disease.

## 5. Conclusions

In conclusion, this study identified *Lasiodiplodia theobromae* as a new causal agent of rubber tree stem gummosis in China, based on pathogenicity assays, morphological characterization, and multi-locus phylogenetic analyses. To our knowledge, this is the first report of *L. theobromae* causing stem gummosis on rubber tree in China. Field experiment showed that the integration of fungicide application (Bordeaux mixture trunk coating + root irrigation using citric acid–copper 6.4% + chelated copper-ammonium 15% at both 0.1% and 0.2% concentration) showed potential for disease control, offering new strategies for management. The findings of this study can provide valuable information for future monitoring and management strategies for this disease.

## Figures and Tables

**Table 1:** *Lasiodiplodia* species with GenBank accession numbers used for phylogenetic analysis.

Species	Isolate	Location	GenBank Accession Number		
			ITS	*TEF1-α*	*TUB2*
*Botryosphaeria dothidea*	CMW 8000	South Africa	AY236949	AY236898	AY236927
*L. avicenniae*	CMW 41467	South Africa	KP860835	KP860680	KP860758
*L. chinensis*	CGMCC 3.18061	China	KX499889	KX499927	KX500002
*L. crassispora*	SMD 30.7	South Africa	OL441871	OL441927	OL441983
*L. crassispora*	CMW 13488	Australia	DQ103552	DQ103559	KU887507
*L. euphorbiaceicola*	CMW 33350	South Africa	KU887149	KU887026	KU887455
*L. euphorbiaceicola*	CMW 36231	South Africa	KU887187	KU887063	KU887494
*L. gonubiensis*	CMW 14078	South Africa	AY639594	DQ103567	EU673126
*L. gonubiensis*	CBS 115812	South Africa	KF766191	DQ458877	KU887512
*L. parva*	CBS 494.78	Portugal	EF622084	EF622064	EU673114
*L. parva*	CBS456.78	Portugal	EF622083	EF622063	KU887523
*L. pseudotheobromae*	CBS 116459	Portugal	EF622077	EF622057	EU673111
*L. pseudotheobromae*	JFRL 03-1149	China	OQ804427	OQ818099	OQ818102
*L. rubropurpurea*	WAC 12536	Australia	DQ103554	DQ103572	KP872425
*L. sterculiae*	CBS 342.78	Netherlands	KX464140	KX464634	KX464908
*L. theobromae*	CBS 164.96	Portugal	AY640255	AY640258	EU673110
*L. theobromae*	CAA 006	Portugal	DQ458891	DQ458876	DQ458859
*L. venezuelensis*	CBS 129753	USA	JX545100	JX545120	JX545140
*L. vitis*	CBS 124060	Netherlands	KX464148	KX464642	KX464917

## Data Availability

Sequence data from this article can be found in GenBank at https://www.ncbi.nlm.nih.gov/datasets/genome/ (accessed on 25 November 2023) with the accession numbers listed in the Results Section. All other relevant data are within the paper.

## Supplementary Materials

The following supporting information can be downloaded at: https://www.mdpi.com/article/10.3390/microorganisms13071586/s1, Table S1. The ITS, *TEF-1α* and *TUB2* sequences of *Lasiodiplodia Pseudotheobromae* amplified using the primer ITS1/ITS4 [25], EF1-688 (F)/EF1-1251(R) [26], and Bt2a/Bt2b [27].
