# Supplementary material for: Morphological and Molecular Characterization of Lasiodiplodia theobromae Causing Stem Gummosis Disease in Rubber Trees and Its Chemical Control Strategies
Source: Microorganisms. 2025 Jul 5;13(7):1586. doi: 10.3390/microorganisms13071586 (PMC12299842; doi:10.3390/microorganisms13071586)
Supplement: Supplementary file 1 [file microorganisms-13-01586-s001.zip › microorganisms-3695382-supplementary.pdf]

Table S1. The ITS, *TEF1-α* and *TUB2* sequences of *Lasiodiplodia theobromae* amplified using the primer ITS1/ITS4[White et al 1990], EF1-688 (F)/ EF1-1251(R) [Alves et al 2008], and Bt2a/ Bt2b [Glass and Donaldson 1995].

No.	Gene	Isolate	Sequences
1	ITS (519bp)	J2	TTCCGTAGGTGAACCTGCGGGAGGATCATTACCGAGTTTTCGAGCTCCGG CTCGACTCTCCCACCCTTTGTGAACGTACCTCTGTTGCTTTGGCGGCTCC GGCCGCCAAAGGACCTTCAAACTCCAGTCAGTAAACGCAGACGTCTGAT AAACAAGTTAATAAACTAAAACTTTCAACAACGGATCTCTTGGTTCTGGC ATCGATGAAGAACGCAGCGAAATGCGATAAGTAATGTGAATTGCAGAATT CAGTGAATCATCGAATCTTTGAACGCACATTGCGCCCCTTGGTATTCCGG GGGGCATGCCTGTTCGAGCGTCATTACAACCCTCAAGCTCTGCTTGGAAT TGGGCACCGTCCTCACTGCGGACGCGCCTCAAAGACCTCGGCGGTGGCT GTTCAGCCCTCAAGCGTAGTAGAATACACCTCGCTTTGGAGCGGTTGGCG TCGCCCGCCGGACGAACCTTCTGAACTTTTCTCAAGGTTGACCTCGGATC AGGTAGCAGCCCCCCAATTCT
2	*TEF1-α* (520bp)	J2	GCTACAAGTGCGGCGGTATTGACAAGCGTACCATTGAGAAGTTCGAGAA GGTCCGTGCACGCATGTCGTTTTTTAACCCCTCTCGACTTCGGCGCTGCA GCGCTGCGGCGCACAGTCCGCCTTATCGCTTTGGTGAGGGGCATTTTTTC GTGGTGGGGTTTGGCCCGCGCTAGCCTCGTCTGGGTTCGGCAAAATGAC CGCACTTGGTTTTTTTGCGACCGGCGTCTGGCCGACGCGCCTCCCCACTA GCGAAAAATGCTCTGACCACTCATGTACCGTCGTACGAGCAAAGGCTAA CGCGCCCCACTACAGGAAGCCGCTGAGCTCGGCAAGGGTTCCTTCAAGT ACGCCTGGGTTCTTGACAAGCTCAAGGCCGAGCGTGAGCGTGGTATCAC CATCGACATTGCCCTCTGGAAGTTCGAGACCCCGAAGTACTATGTCACCG TCATCGACGCCCCCGGTCACCGTGACTTCATCAAGAACATGATCACTGGT ACCTCGCAGGCCGACTGCGCCATCC
3	*TUB2* (407bp)	J2	CGGTGCTGCTTTCTGGTTTGTTGCCAAAACACTCCTGCTCCTGCGCCCCC CGCTGACGGAAGCGACACCATAGGCAGACCATCTCCGGCGAGCACGGCC TGGATGGCTCCGGTGTGTAAGTGTGCGCCTTCTCCGCCGCGCATGGCAAT CGCTGACCTGTAGCAGCTACAATGGCACTTCGGACCTCCAACTGGAGCG CATGAACGTCTACTTCAACGAGGTACTCTCTCCATAATTAGACAAACACG TAAAGTATGGCAATCTTCTGAACGCGCAGCAGGCGTCCAACAACAAGTA CGTTCCTCGTGCTGTCCTCGTCGACCTCGAGCCCGGCACCATGGATGCCG TCCGCGCCGGCCCCTTCGGCCAGCTCTTCCGCCCCGACAACTTCGTCTTC GGCCAGTCTG
